# Prenatal fine particulate exposure associated with reduced childhood lung function and nasal epithelia GSTP1 hypermethylation: Sex-specific effects

**DOI:** 10.1186/s12931-018-0774-3

**Published:** 2018-04-27

**Authors:** Alison G. Lee, Blake Le Grand, Hsiao-Hsien Leon Hsu, Yueh-Hsiu Mathilda Chiu, Kasey J. Brennan, Sonali Bose, Maria José Rosa, Kelly J. Brunst, Itai Kloog, Ander Wilson, Joel Schwartz, Wayne Morgan, Brent A. Coull, Robert O. Wright, Andrea A. Baccarelli, Rosalind J. Wright

**Affiliations:** 1 Division of Pulmonary, Critical Care and Sleep Medicine, Icahn School of Medicine at Mount Sinai, 1236 Park Avenue, First Floor, New York, NY 10029 USA; 2 Department of Environmental Medicine and Public Health, Icahn School of Medicine at Mount Sinai, New York, NY USA; 3 Department of Pediatrics, Kravis Children’s Hospital, Icahn School of Medicine at Mount Sinai, New York, NY USA; 4 Department of Environmental Health Sciences, Columbia University Mailman School of Public Health, New York, New York, USA; 5 Department of Environmental Health, University of Cincinnati College of Medicine, 160 Panzeca Way, Cincinnati, OH USA; 6 Department of Geography and Environmental Development, Faculty of Humanities and Social Sciences, Ben-Gurion University of the Negev, Beer Sheva, Israel; 7 Department of Statistics, Colorado State University, Fort Collins, CO USA; 8 Department of Environmental Health, Harvard T.H. Chan School of Public Health, Boston, MA USA; 9 Department of Pediatrics, University of Arizona, Tucson, Arizona USA; 10 Department of Biostatistics, Harvard T.H. Chan School of Public Health, Boston, MA USA

## Abstract

**Background:**

In utero exposure to particulate matter with an aerodynamic diameter of less than 2.5 μm (PM_2.5_) has been linked to child lung function. Overlapping evidence suggests that child sex and exposure timing may modify effects and associations may be mediated through glutathione S-transferase P1 (GSTP1) methylation.

**Methods:**

We prospectively examined associations among prenatal PM_2.5_ exposure and child lung function and GSTP1 methylation in an urban pregnancy cohort study. We employed a validated satellite-based spatiotemporally resolved prediction model to estimate daily prenatal PM_2.5_ exposure over gestation. We used Baysian distributed lag interaction models (BDLIMs) to identify sensitive windows for prenatal PM_2.5_ exposure on child lung function and nasal epithelia GSTP1 methylation at age 7 years, and to examine effect modification by child sex.

**Results:**

BDLIMs identified a sensitive window for prenatal PM_2.5_ exposure at 35–40 weeks gestation [cumulative effect estimate (CEE) = − 0.10, 95%CI = − 0.19 to − 0.01, per μg/m^3^ increase in PM_2.5_] and at 36–40 weeks (CEE = − 0.12, 95%CI = − 0.20 to − 0.01) on FEV_1_ and FVC, respectively, in boys. BDLIMs also identified a sensitive window of exposure at 37–40 weeks gestation between higher prenatal PM_2.5_ exposure and increased GSTP1 percent methylation. The association between higher GSTP1 percent methylation and decreased FEV1 was borderline significant in the sample as a whole (*β* = − 0.37, SE = 0.20, *p* = 0.06) and in boys in stratified analyses (*β* = − 0.56, SE = 0.29, *p* = 0.05).

**Conclusions:**

Prenatal PM_2.5_ exposure in late pregnancy was associated with impaired early childhood lung function and hypermethylation of GSTPI in DNA isolated from nasal epithelial cells. There was a trend towards higher GSTP1 percent methylation being associated with reduced FEV1. All findings were most evident among boys.

## Background

The importance of early life lung development on future respiratory health is well established [1, 2]. Understanding environmental exposures and mechanisms that lead to and maintain this early predisposition is key to identifying children at increased risk of future respiratory disease and, ultimately, reducing chronic pulmonary disease morbidity and mortality. Genetic variants and early life factors including tobacco smoke, birth weight, gestational age, and asthma are associated with reduced lung function over the life course, however these factors account for a relatively small proportion of the risk suggesting that unidentified factors remain.

Animal models have highlighted the need to understand PM effects on lung development starting prenatally [3, 4]. Recent human data corroborates the association between in utero PM_2.5_ exposure and asthma risk in children with an increased focus on sensitive windows for exposure effects [5]. To our knowledge, only one study has reported associations between prenatal exposure to PM_2.5_ and reduced child lung function, however exposure was measured at a single time point and extrapolated over pregnancy and thus could not flexibly assess sensitive windows of effect [6]. Because the well-orchestrated processes involved in programming lung growth and development over gestation do not necessarily occur within clinically defined trimesters, research that allows flexibility in identifying sensitive windows may be particularly informative [7]. Further, prior research suggests that boys may be more vulnerable to the effects of prenatal environmental exposures, including air pollution exposure [5], perhaps mediated through increased vulnerability to oxidative stress.

Epigenetic programing, including DNA methylation, starting during fetal life is one pathway by which environmental factors may influence gene expression thus programing future disease risk [8, 9]. Global and gene-specific methylation is altered in response to prenatal exposures and these changes appear stable in early childhood [10, 11]. For example, an epigenome-wide study of cord blood DNA methylation in children prenatally exposed to nitrogen dioxide (NO_2_) demonstrated differential methylation of genes involved in mitochondrial function [12], and a study examining exposure to PM_10_ averaged over trimesters in relation to loci specific methylation found that early pregnancy PM_10_ exposure was associated with placental DNA methylation of LINE1 and HSD11B2 [13].

Particulate matter is a strong oxidant able to generate reactive oxygen species (ROS); oxidative stress pathways are believed to be central in the association between air pollution exposure and respiratory outcomes [14, 15]. The glutathione S-transferase (GST) superfamily, specifically the P1 isoform (GSTP1), is expressed in the respiratory tract and functions in oxidant defenses, xenobiotic metabolism, and detoxification of hyperperoxides; GSTP1 variants have been found to increase susceptibility to tobacco smoke and air pollution in several studies [16–18]. GSTP1 CpG promoter hypermethylation may inactivate GSTP1 [19], thereby reducing cellular detoxification capabilities and increasing susceptibility to oxidative stress [20].

The airway epithelium is an important driver of pulmonary disease – for example, the airway epithelium regulates inflammatory responses in asthma [21]. Lower airway, or bronchial, epithelial cell sampling requires invasive procedures (e.g., bronchoscopy or lung tissue biopsy), which are not suitable for healthy children. As nasal epithelial cells (NECs) are surrogates for bronchial epithelial cells and more readily accessible, it has been proposed that examination of associations between environmental pollutants and epigenetic changes in DNA isolated from NECs may provide insight into the mechanisms that underlie associations between PM_2.5_ and airway diseases [22–24].

We examined the impact of prenatal PM_2.5_ exposure on children’s lung function in an urban, ethnically mixed longitudinal pregnancy cohort. We employed Bayesian distributed lag interaction models (BDLIMs) to estimate windows of sensitivity between prenatal PM_2.5_ exposure and children’s pulmonary function measured at age 7 years and to examine effect modification by child sex. We also examined associations between prenatal PM_2.5_ exposure and GSTP1 percent methylation in DNA from NECs isolated in children at time of spirometry. Finally we explored associations between GSTP1 percent methylation and children’s lung function measures. We hypothesized that children born to mothers with higher PM_2.5_ exposure would have lower spirometry outcomes and hypermethylation of NEC GSTP1 at age 7 years, relative to those born to mothers with low PM_2.5_ exposure. We posited that the highest levels of GSTP1 percent methylation would be associated with greater reductions in spirometry measures. We hypothesized that boys would be particularly vulnerable.

## Methods

### Study participants

Subjects were from the Asthma Coalition on Community, Environment and Social Stress (ACCESS) project, an urban ethnically-diverse pregnancy cohort designed to examine the effects of environmental exposures on childhood respiratory outcomes [25]. From August 2002 through January 2007, *n* = 500 pregnant women were recruited from two Boston hospitals and affiliated health centers at 28.4 ± 7.9 weeks gestation and 455 gave birth to a live singleton infant. Of those approached and eligible, 78% were enrolled; no significant differences in education, race/ethnicity, or income were observed in those who enrolled versus those who were not enrolled. A subset (230 of 375) of children actively followed at age 6.99 ± 0.89 years participated in a pulmonary function visit, during which time NECs were also collected. Procedures were approved by human studies committees at the Brigham and Women’s Hospital and Boston Medical Center and written consent was obtained from all mothers and assent was obtained for children age ≥7 years.

### Daily prenatal PM_2.5_ levels

Maternal residence was geocoded at baseline and updated throughout gestation if the subject moved; geocoded addresses were used to estimate residence-specific daily prenatal PM_2.5_ exposure over the pregnancy, as detailed previously [26]. Briefly, a novel spatiotemporal model estimated daily high resolution PM_2.5_ by regressing daily surface PM_2.5_ measurements, taken from the U.S. Environmental Protection Agency Air Quality System and Interagency Monitoring of Protected Visual Environments Network, with daily aerosol optical depth measurements, land-use terms (elevation, distance to major roads, percent open space, point emissions, and area emissions), and meteorological variables (temperature, wind speed, visibility). The model linked moderate resolution imaging spectroradiometer satellite-derived aerosol optical depth measurements at a 1 km × 1 km spatial resolution and ground measurement covering the Northeast USA were calibrated on a daily basis and validated with robust out of sample 10-fold cross-validation. The mean cross-validated R^2^ for the New England sub-region that includes the greater Boston area included in this study was 0.88. To reduce noise created by the day-to-day PM_2.5_ variation, women’s gestational PM_2.5_ exposure estimates were calculated by averaging daily predictions over each week of the pregnancy for these analyses.

### Pulmonary function testing

Research assistants trained by an experienced pediatric respiratory therapist and pulmonologists (WJM, RJW) on our team measured child height, weight, and lung function with over-reading performed for all spirometry tests to ensure quality control. Height was measured to the nearest 0.1 cm using a stadiometer and weight was measured to the nearest 0.1 kg using an electronic scale. Spirometry was performed in participant homes with a portable MedGraphics™ laptop supported spirometer, which displays real-time flow-volume plots to facilitate testing. Testing procedures met American Thoracic Society guidelines [27, 28] with techniques modified for children ≤8 years of age [29, 30]. Subjects without acute respiratory symptoms for ≥3 weeks were eligible for testing. Short-acting beta-agonists, atropinics and theophylline preparations were withheld for 4 h and long-acting beta-agonists for 12 h before testing. Forced vital capacity (FVC, milliliters), forced expiratory volume in 1 s (FEV_1_, milliliters), and forced expiratory flow between 25 and 75% of the FVC (FEF_25–75_, milliliters per second) were recorded from a minimum of 3 (no more than 8) maneuvers. Lung function measures, height and weight were all approximately normally distributed. Raw FEV_1_, FVC, FEF_25–75_ and FEV_1_/FVC values were adjusted for age, sex, height, and race/ethnicity using multivariable regression, and then converted to z-scores with a mean of 0 and a standard deviation of 1 to describe each child’s position relative to that of other individuals in the distribution, as done in previous studies [31, 32].

### GSTP-1 percent methylation

Epithelial cells were collected from the anterior nares. Briefly, the child was seated in a chair with head tilted back. Each nostril was cleaned with a cotton swab wet in saline solution. A 1 mm Microinvasive Boston specimen brush was inserted into each nostril until the tip was immediately inferior to the nasal bone and vigorously rotated along the nares avoiding the nasal septum. Notably, prior work by our group has demonstrated methylation concordance (R^2^ = 0.93) between inferior turbinate and anterior nares cells, providing an acceptable and safer, less-invasive sampling procedure for young children [33]. The brushes were immediately submersed in 6 mL of 2% acetylcysteine-saline solution. Samples were incubated at room temperature with gentle shaking for 30 min, then the brush was removed and the solution was centrifuged at 4500 rpm for 10 min. DNA was extracted from the cell pellet using the Promega Maxwell^R^ 16 Buccal Swab LEV DNA Kit following manufacturer instructions and stored at − 80 °C prior to bisulfite conversion.

### GSTP1 methylation analyses

The nasal cell DNA was bisulfite converted using the EZ DNA Methylation-Gold Kit (Zymo Research, CA, USA) according to the manufacturer’s protocol. Polymerase chain reaction (PCR) and pyrosequencing was used to quantify the percentage methylation at each site of interest. Control samples were used to verify bisulfite conversion efficiency. The measured degree of methylation is presented as the percentage of methylated cytosines divided by the sum of methylated and unmethylated cytosines (%5mC). Pyrosequencing primers were designed not to overlap with any single-nucleotide polymorphism or repeated elements [34]. The forward PCR primer sequence was TTTGGGAAAGAGGGAAAGGT and the reverse 5′ end biotin labeled primer was AACCTTATAAAAATAATCCC. PCR cycling conditions were a 5 min hold at 95 °C followed by 95 °C for 45 s, 50 °C for 45 s, and 72 °C for 45 s for 45 cycles, followed by a 5 min hold at 72 °C. PCR products were purified and sequenced on a Q96 MD pyrosequencing system (Qiagen), as previously described [35]. The sequencing primer sequence was AGAGGGAAAGGTTTTTT and the sequence entry was CGGTTAGTTGCGCGGCGATTTCGGGGATTTTAGGGCGTTTTTTTGCGGTCGACGTTCGGGGTGTAGCGGTCGTCGGGGTTGGGGTCGGCGGGAGTTCGCGGGATT. Seventeen CpG sites were analyzed on chromosome 11 (GRCh37/hg19).

### Covariates

Maternal race, age at enrollment, and education were obtained through questionnaires. Mothers who reported smoking at baseline or in the third trimester of pregnancy were classified as smokers; postnatal secondhand tobacco smoke exposure was classified based on maternal-reported smoking or report of others smoking in the home at each postpartum interview. Maternal-reported clinician-diagnosed asthma was determined at 3-month intervals for the first 24 months of life and annually thereafter until age 6 years. Mothers were asked, “Has a doctor or nurse ever said that your child had asthma?”. The majority of children received a diagnosis of asthma after 3 years of age (78.7%) [5].

### Statistical analysis

Of the 230 children recruited for the spirometry visit, 211 (92%) provided acceptable spirometry. Complete data on prenatal daily PM_2.5_ levels was available in 192 (91%); of these, 171 were born full-term (born at ≥37 weeks gestation) and were included in the analysis examining associations between prenatal PM_2.5_ and spirometry outcomes. In this analytic sample, 131/171 (76.6%) also had adequate nasal cell DNA for GSTP1 assays.

In order to identify sensitive windows for the effects of prenatal PM_2.5_ in relation to spirometry outcomes and GSTP1 DNA methylation, as well as effect modification by sex, we implemented Bayesian distributed lag interaction models (BDLIMs) as detailed previously [7]. Using a BDLIM, we estimated the time-varying association for each participant’s weekly exposures throughout the gestational period and z-scores of spirometry outcomes. BDLIM extends the traditional constrained distributed lag model framework that identifies sensitive windows [36], and also accounts for within window effects and tests for effect modifications. Significant sensitive exposure windows were identified as weeks during pregnancy with a statistically significant association.

We first conducted BDLIM in the overall sample and then examined effect modification by child’s sex. BDLIM partitions the distributed lag function into two components: 1) the weights that identify sensitive windows of susceptibility, and 2) the coefficients that identify the magnitude of the within-window effects. Thus, BDLIM is able to detect whether boys and girls have either the same, or different, sensitive windows (weights) and magnitude of within-window effects (effects). In other words, models with 4 types of patterns (i.e., same weight and same effect, same weight and different effects, different windows and same effect, different windows and different effects) were tested to determine whether the associations between weekly PM_2.5_ and the outcomes were modified by sex. Subsequently, in addition to estimating the time-varying associations, BDLIM is also able to estimate the cumulative effects of PM_2.5_ exposure over pregnancy that account for both sensitive windows and within-window effects corresponding to each sex. Deviance information criterion (DIC) was used to determine the best fitting model with optimal number of knots and whether the weights and effects are different across sex.

For analysis on spirometry outcomes (i.e., FEV_1_, FVC, FEF_25–75_, FEV_1_/FVC), in addition to the covariates adjusted in z-scores including age, sex, height and race/ethnicity, we also adjusted for maternal age and education in the main analysis. Analysis on GSTP1 percent methylation were adjusted for child’s age, sex, race/ethnicity, maternal age and education.

The distribution of GSTP1 percent methylation was right-skewed (median 2.26, IQR 1.56–3.02). We hypothesized that high percent methylation (hypermethylation) would be associated with impaired lung function. Therefore, GSTP1 percent methylation was a priori dichotomized into high [fourth quartile; ≥3.02 (*n* = 33)] versus low [< 3.02 (*n* = 98)]. Multivariable linear regression models were employed to examine the association between low versus high GSTP1 methylation on PFT z-scores with additional adjustment for maternal age and education. Sex-stratified analyses were also explored. In sensitivity analysis, we also further adjusted for pre- and postnatal smoking, as well as children’s asthma which is likely on the pathway between air pollution and lung function.

BDLIM analyses were implemented in R statistical software (v3.3.1, Vienna, Austria) and descriptive analyses as well as linear regression models were implemented in SAS (v9.4, SAS Institute, Cary, NC).

## Results

Table 1 summarizes participant characteristics. The distribution of covariates among those included in the spirometry analysis and those in the GSTP1 analysis were similar. Most mothers were ethnic minority (predominantly Hispanic, followed by Black), had ≤12 years of education (~ 60%), and never smoked (~ 70%). Average prenatal PM_2.5_ levels were similar for both groups and there were no significant differences in terms of maternal age at enrollment, race/ethnicity, education, pre- and postnatal smoking, and child’s asthma status (Table 1).

**Table 1:** ACCESS participant characteristics

	Included in spirometry analysis		Included in GSTP1 analysis	
	(*n* = 171)		(*n* = 131)	
Prenatal PM_2.5_ level (μg/m^3^; median, IQR) ^a^	10.9	10.2─11.7	11.0	10.2─11.8
Child sex (n, %)				
Girls	82	48.0	63	48.1
Boys	89	52.1	68	51.9
Race/Ethnicity (n, %)				
Black	39	22.8	28	21.4
Hispanic	112	65.5	85	64.9
White/Other	20	11.7	18	13.7
Maternal education (n, %)				
> 12 yrs	57	33.3	53	40.5
≤ 12 yrs	114	66.7	78	59.5
Child age at spirometry measure (yr; mean, SD)	6.9	0.8	6.9	0.8
Maternal age at enrollment (yr; mean, SD)	27.2	5.7	27.6	5.7
Pre- and postnatal smoking status (n, %) ^b^				
Never smoked	122	71.4	95	72.5
Smoked prenatally, but not postnatally	14	8.2	11	8.4
Did not smoke prenatally, but smoked postnatally	24	14.0	16	12.2
Smoked both pre- and postnatally	11	6.4	9	6.9
Child asthma status (n, %)				
No	144	84.2	109	83.2
Yes	27	15.8	22	16.8
Spirometry outcomes				
FEV_1_ raw value (L; mean, SD)	1.44	0.25	1.42	0.25
FVC raw value (L; mean, SD)	1.58	0.29	1.55	0.28
FEV_1_/FVC ratio (mean, SD)	0.92	0.05	0.92	0.05
FEF_25–75_ raw value (L/s; mean, SD)	1.87	0.46	1.88	0.46
z-score of FEV_1_ (mean, SD) ^c^	0.01	0.99	0.00	1.03
z-score of FVC (mean, SD) ^c^	−0.04	0.99	−0.06	1.02
z-score of FEV_1_/FVC ratio (mean, SD) ^c^	0.04	1.00	0.06	0.98
z-score of FEF_25–75_ (mean, SD) ^c^	0.08	0.96	0.07	0.98

^a^Averaged over entire pregnancy

^b^Combination of prenatal maternal smoking and postnatal household smoking status

^c^Adjusted for age, sex, height, race

### Effects of prenatal PM_2.5_ exposure on child spirometry

**Fig. 1:**
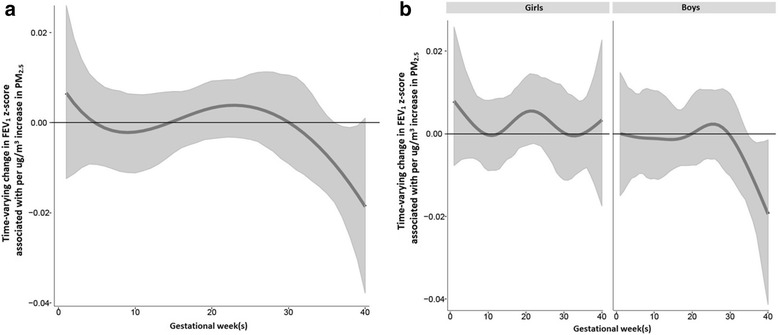
Associations between weekly PM_2.5_ levels over gestation and FEV_1_ z-score. This figure demonstrates the association between PM_2.5_ exposure over pregnancy and FEV_1_ z-scores using a BDLIM assuming week-specific effects, for (**a**) overall sample, and (**b**) interaction by sex. Models were adjusted for maternal age and education, and z-scores were adjusted for child’s age at spirometry test, sex, race/ethnicity, and height. The y-axis represents the change in FEV_1_ z-scores corresponding to a 1 μg/m^3^ increase in PM_2.5_; the x-axis is gestational age in weeks. Solid lines show the predicted change in FEV_1_ z-score. Gray areas indicate 95% confidence intervals (CIs). A sensitive window is identified for the weeks where the estimated pointwise 95% CI (shaded area) does not include zero

In the sample considered as a whole, BDLIMs identified a statistically significant sensitive window of exposure (36–39 weeks gestation) during which children exposed to higher prenatal PM_2.5_ had an increased risk of reduced FEV_1_ z-score, after adjusting for maternal age and education and z-score adjustment for child age, sex, height and weight (Fig. 1a). In sex-stratified analyses, we observed a similar significant sensitive window of prenatal PM_2.5_ exposure at 35–40 weeks gestation in boys whereas no significant sensitive window was seen in girls (Fig. 1b). The results from BDLIMs suggested that the interaction between prenatal PM_2.5_ and sex was attributable to both different sensitive windows and different within-window effects (the normalized posterior density was 0.86 for the model with this assumption, which can be interpreted as a probability that this was the best fitting pattern of effect modification). In order to further assess the time-weighted associations over the entire pregnancy, we also estimated the cumulative effects accounting for identified sensitive windows and within-window associations. The estimated cumulative effect of prenatal PM_2.5_ per 1 μg/m^3^ increase in PM_2.5_ was significant for boys (cumulative effect estimate = − 0.10, 95%CI = − 0.19 to − 0.01), but not in girls (Table 2).

**Table 2:** Estimated cumulative effects of prenatal PM2.5 exposure over gestation: accounting for interaction by sex, sensitive windows and within-window effects identified by BDLIM

Spirometry (z-scores)^a^	Overall			Interaction Model					
				Girls			Boys		
	Cumulative Effect	95% CI		Cumulative Effect	95% CI		Cumulative Effect	95% CI	
FEV_1_	− 0.05	− 0.13	0.02	0.10	0.00	0.18	*− 0.10*	*− 0.19*	*− 0.01*
FVC	*− 0.09*	*− 0.17*	*− 0.02*	0.03	− 0.05	0.14	*− 0.12*	*− 0.20*	*−0.01*
FEV_1_/FVC	0.03	−0.05	0.09	0.02	−0.04	0.11	0.02	−0.04	0.10
FEF_25–75_	0.03	−0.05	0.09	0.08	−0.01	0.17	0.00	−0.07	0.07

^a^Models were adjusted for maternal age and education, and z-scores were adjusted for child’s age at spirometry test, sex, height, race/ethnicity; corresponding to per 1 μg/m^3^ increase in PM_2.5_

**Fig. 2:**
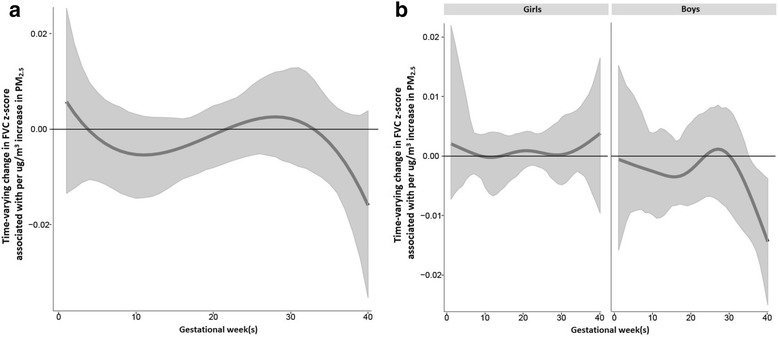
Associations between weekly PM_2.5_ levels over gestation and FVC z-score. This figure demonstrates the association between PM_2.5_ exposure over pregnancy and FVC z-scores using a BDLIM assuming week-specific effects, for (**a**) overall sample, and (**b**) interaction by sex. Models were adjusted for maternal age and education, and z-scores were adjusted for child’s age at spirometry test, sex, race/ethnicity, and height. The y-axis represents the change in FVC z-scores corresponding to a 1 μg/m^3^ increase in PM_2.5_; the x-axis is gestational age in weeks. Solid lines show the predicted change in FVC z-score. Gray areas indicate 95% confidence intervals (CIs). A sensitive window is identified for the weeks where the estimated pointwise 95% CI (shaded area) does not include zero

When considering the sample as a whole, the estimated cumulative effect of PM_2.5_ exposure across pregnancy on FVC, accounting for time-varying effects determined by BDLIMs was also significant (cumulative effect estimate = − 0.09 per 1 μg/m^3^ increase in PM_2.5_, 95%CI = − 0.17 to − 0.02; Table 2). BDLIMs did not identify a statistically significant sensitive window of exposure between prenatal PM_2.5_ and FVC in the overall sample, however models did identify a sensitive window when also considering infant sex (Fig. 2a). Specifically, the BDLIM accounting for sex demonstrated that boys born to mothers with increased prenatal PM_2.5_ exposure at 36–40 weeks gestation were at increased risk of having reduced FVC z-scores (Fig. 2b), and the estimated cumulative effect was also statistically significant in boys (cumulative effect estimate = − 0.12 per 1 μg/m^3^ increase in PM_2.5_, 95%CI = − 0.20 to − 0.01; Table 2). No significant window or cumulative effect was found among girls.

For the FEV_1_/FVC ratio, BDLIMs identified a statistically significant sensitive window of exposure to prenatal PM_2.5_ at 34–36 weeks gestation and reduced FEV_1_/FVC z-score. However, the overall cumulative effect was not significant (Table 2), and no significant effect modification by child sex was found. For FEF_25–75_, BDLIMs did not identify a statistically significant sensitive window of exposure between prenatal PM_2.5_ and reduced FEF_25–75_ z-score; the estimated cumulative effect of PM_2.5_ exposure across pregnancy on FEF_25–75_ z-score was not significant (Table 2), nor was effect modification by child sex.

### Effect of prenatal PM_2.5_ exposure on GSTP1 percent methylation

**Fig. 3:**
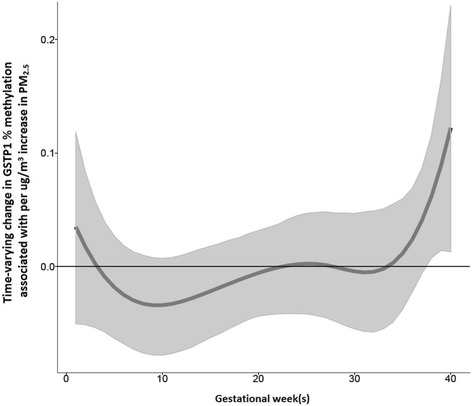
Associations between weekly PM_2.5_ levels over gestation and GSTP1 percent methylation. This figure demonstrates the association between PM_2.5_ exposure over pregnancy and GSTP1 DNA methylation using a BDLIM assuming week-specific effects. The model was adjusted for maternal age, education, child’s age at spirometry test, sex, and race/ethnicity. The y-axis represents the change in GSTP1 percent methylation corresponding to a 1 μg/m^3^ increase in PM_2.5_; the x-axis is gestational age in weeks. Solid lines show the predicted change in GSTP1 percent methylation. Gray areas indicate 95% confidence intervals (CIs). A sensitive window is identified for the weeks where the estimated pointwise 95% CI (shaded area) does not include zero

We found a statistically significant sensitive window of exposure between prenatal PM_2.5_ at 37–40 weeks gestation and increased GSTP1 percent methylation (Fig. 3), although the overall estimated cumulative effect across pregnancy was not significant (cumulative effect estimate =0.01 per 1 μg/m^3^ increase in PM_2.5_, 95%CI = − 0.78 to 0.76). No sex-specific effects were identified.

### Associations between GSTP1 percent methylation and child spirometry

In the sample as a whole, high percent methylation was associated with decreases in FEV_1_ (z-score = − 0.38, *p* = 0.06) and FEF_25–75_ (z-score = − 0.38, *p* = 0.05), adjusting for maternal age and education (Table 3, Model 1) although associations did not quite reach statistical significance at the *p* < 0..05 level. Sensitivity analysis with additional adjustment for child asthma and tobacco smoke exposure did not significantly change the point estimates (Table 3, Models 2 and 3). In sex-stratified analyses, associations between high GSTP1 percent methylation and reduced FEV1 (z-score = − 0.56, p = 0.05) and FEF_25–75_ (z-score = − 0.54, p = 0.06) were only seen among boys and associations were again borderline significant (Table 4). Interaction terms did not approach significance.

**Table 3:** Associations between GSTP1%methylation^a^ (%m) and childhood PFT z-scores^b^: Linear regression

Spirometry (z-scores)^a^	Univariate Model			Multivariable-adjusted Models^c^								
				Model 1^d^			Model 2^e^			Model 3^f^		
	β	s.e.	*p*	Β	s.e.	*p*	β	s.e.	*p*	β	s.e.	*p*
*FEV*_*1*_												
GSTP1%m low	Ref	–	–	Ref	–	–	Ref	–	–	Ref	–	–
GSTP1%m high	−0.38	0.20	0.06	−0.37	0.20	0.06	−0.40	0.20	0.05	−0.41	0.20	0.04
*FVC*												
GSTP1%m low	Ref	–	–	Ref	–	–	Ref	–	–	Ref	–	–
GSTP1%m high	−0.20	0.20	0.32	−0.19	0.21	0.36	−0.21	0.21	0.29	−0.22	0.21	0.27
*FEV*_*1*_ */FVC*												
GSTP1%m low	Ref	–	–	Ref	–	–	Ref	–	–	Ref	–	–
GSTP1%m high	−0.27	0.19	0.16	−0.27	0.19	0.16	−0.27	0.20	0.16	−0.27	0.20	0.16
*FEF*_*25–75*_												
GSTP1%m low	Ref	–	–	Ref	–	–	Ref	–	–	Ref	–	–
GSTP1%m high	−0.37	0.19	0.05	−0.38	0.19	0.05	−0.40	0.19	0.04	−0.39	0.19	0.04

^a^GSTP1 average methylation ≥3.02 [n = 33 (25%)] vs < 3.02; dichotomized around 4th quartile

^b^PFT z-scores adjusted for age, sex, height, race

^c^Multivariable-adjusted linear regressions (Models 1–3) predicting PFT z-scores (dependent variables)

^d^Model 1 additionally adjusted for maternal age and education

^e^Model 2 (sensitivity model) additionally adjusted for child asthma

^f^Model 3 (sensitivity model) additionally adjusted for child asthma and tobacco smoke exposure

**Table 4:** Sex-stratified associations between GSTP1%methylation^a^ (%m) and childhood PFT z-scores^b^: Linear regression^c^

Spirometry (z-scores)	Girls (low *n* = 65; high, *n* = 16)			Boys (low *n* = 67, high *n* = 17)			p-interaction
	Β	s.e.	*p*	β	s.e.	*p*	
*FEV*_*1*_							
GSTP1%m low	Ref	–	–	Ref	–	–	
GSTP1%m high	−0.26	0.29	0.38	−0.56	0.29	0.05	0.37
*FVC*							
GSTP1%m low	Ref	–	–	Ref	–	–	
GSTP1%m high	−0.14	0.28	0.61	−0.31	0.31	0.32	0.53
*FEV*_*1*_ */FVC*							
GSTP1%m low	Ref	–	–	Ref	–		
GSTP1%m high	−0.15	0.23	0.51	−0.38	0.33	0.26	0.67
*FEF*_*25–75*_							
GSTP1%m low	Ref	–	–	Ref	–	–	
GSTP1%m high	−0.26	0.27	0.34	−0.54	0.28	0.06	0.54

^a^GSTP1 average methylation ≥3.02 vs < 3.02; dichotomized around 4th quartile

^b^PFT z-scores adjusted for age, sex, height, race

^c^Multivariable-adjusted linear regressions additionally adjusted for maternal age and education predicting PFT z-scores (dependent variables)

## Discussion

This is the first prospective study to combine advanced statistical modeling with highly resolved ambient PM_2.5_ exposure estimates to objectively determine susceptible windows of exposure between prenatal PM_2.5_ exposure and early childhood lung function. Children born to mothers exposed to higher PM_2.5_ in late pregnancy (≥ 35 weeks gestation) were at increased risk for impaired lung function with male children in particular being at increased risk. Higher prenatal PM_2.5_ exposure was also associated with increased GSTP1 methylation in children’s nasal cell DNA with models showing a similar window of susceptibility (37–40 weeks gestation). Linear regression models showed an inverse relationship between increased GSTP1 DNA methylation and reduced lung function, specifically amongst boys. These findings combined with prior literature showing that GSTP1 methylation may decrease GSTP1 expression and increase susceptibility to oxidative stress [19], suggest that epigenetic regulation of GSTP1 may be one pathway underlying the association between prenatal PM_2.5_ exposure and reduced lung function outcomes in early childhood.

A more refined temporal understanding of the impact of prenatal PM_2.5_ exposure on lung function can better delineate underlying mechanisms. Only one prior study has tried to address effects of exposure timing across pregnancy in this context. Morales and colleagues investigated associations between average trimester benzene and nitrogen dioxide (NO_2_) exposure and child lung function at age 4.5 years, demonstrating an inverse relationship between second trimester benzene and NO_2_ exposures and lung function [37]. Our study adds to the literature by investigating a different pollutant, PM_2.5_. Further, studies considering relatively arbitrary assignment of exposure windows (e.g., averaged over pregnancy or within clinically defined trimesters) rather than being grounded in an understanding of well-orchestrated and timed developmental processes relevant to lung development progressing over gestation may introduce bias or miss effect altogether [7]. By using data-driven methods that incorporate both current and prior gestational exposure, we were able to examine associations between prenatal PM_2.5_ exposure and child lung function and identify an unbiased estimate of the window of susceptibility on lung function. Results suggest that the effect of PM_2.5_ on future child lung function largely occurs during the saccular and alveolar phases of lung development. During these phases, the lung undergoes rapid growth and remodeling, including secondary septation of primitive saccules into alveoli, to support efficient gas exchange [38]. Alveologenesis is characterized by proliferation of alveolar type II (ATII) cells, stem cells for alveolar type I cells that line the alveolar surface and produce surfactant. Concurrent elastogenesis spatially instructs future bud formation and it, along with angiogeneisis, is essential to alveolar septation. While our study is the first prospective human study to identify a specific window of susceptibility to prenatal PM_2.5_ for lung function, mouse models of prenatal PM_2.5_ on lung function similarly found that pups exposed to in utero PM_2.5_ had impaired lung function and significantly altered alveolar structure and elastic properties [4].

Lung development and subsequent function is determined by a large number of biological pathways. While heritable genetics play a role, the largest genome-wide association study suggests that 26 loci and > 100 variants may explain 7.5% of FEV1/FVC variance and 3.4% of FEV1 variance [39] highlighting the need to consider gene by environment interactions. Environmental exposures can induce epigenetic modifications, including DNA methylation, that alter gene expression without changing the underlying genetic code [20]. Indeed, one point of epigenome establishment is during the prenatal period and evidence suggests that these changes may persist over the lifecourse or even through generations [10, 12, 40, 41].

Air pollution exposure generates reactive oxygen species (ROS) and the resulting oxidant imbalance is thought to be a central pathway mediating the association between air pollution exposure and impaired lung function [42, 43]. GSTP1 controls enzymes involved in the detoxification of ROS [44] - GSTP1 variants are more susceptible to the health effects of air pollution, including lung function, [45] and GSTP1 methylation is associated with oxidative stress [46]. The developing fetus is especially prone to oxidative injury, as antioxidant defenses remain immature through pregnancy; male fetuses may be at even increased risk. In vitro work exposing ATII cells to PM_2.5_ demonstrates an increase in ROS with subsequent change in ATII phenotype [47]. Pups of pregnant rats exposed to PM_2.5_ during gestation demonstrate impaired lung function with alveolar destruction and thickened alveoli septum demonstrated on histology; maternal PM_2.5_ exposure correlated with increasing levels of pup oxidative stress and markers of oxidative stress in pup lung tissue [48]. While our study demonstrates that prenatal PM_2.5_ is associated with increased GSTP1 methylation during the same window of sensitivity as reduced lung function and that elevated GSTP1 methylation is associated with reduced lung function, future studies with larger sample size are needed to corroborate these findings and to more formally examine mediation.

We note several strengths to our study. We prospectively captured prenatal environmental exposures, a number of important confounders and covariates, and lung function - an objective measure of respiratory outcomes in early childhood - an urban cohort largely composed of ethnic minorities at increased risk for ambient air pollution exposure and impaired lung function. We assessed prenatal PM_2.5_ exposure using a validated hybrid spatiotemporal LUR model incorporating highly-resolved satellite-derived AOD measures. We then objectively identified susceptible windows of exposure for PM_2.5_ on child lung function by using data-driven, advanced statistical techniques to more objectively determine sensitive windows of effect. Also, this is the first study to combine these methods with gene-specific methylation to explore mechanisms mediating the association between prenatal air pollution and child lung function. Also, while studies indicate that tobacco smoke exposure is a major contributor to indoor air pollution in inner-city homes [49, 50], variations in indoor source particles (e.g., from smoking and/or cooking sources) are largely uncorrelated with variations in outdoor source particles, which is the main exposure considered herein [51]. Thus, while particles of indoor origin are an important predictor of fetal development in and of themselves, they are unlikely to confound associations between ambient particulate matter and child lung function. Indeed, investigators have begun to conceptualize tobacco smoke exposure as an effect modifier rather than a confounder in this context [52]. Observed effects remained significant even in sensitivity analyses adjusting for prenatal maternal smoking and second-hand postnatal tobacco smoke exposure, another major determinant of childhood lung function [53].

We also acknowledge limitations. While we adjust for several confounding factors, we did not have data on dietary or other environmental factors, such as temperature, which may co-vary with PM_2.5_. However, as with smoking exposures, maternal diet and temperature are increasingly conceptualized as effect modifiers in the context of health effects of particulate matter rather than confounders [54, 55]. This should be explored in future studies. Our epigenetic analyses involve specimens collected at the time of lung function testing; future studies with serially collected airway epithelial samples will allow formal mediation analyses and expand our understanding of the influence of prenatal environmental exposures on, and stability of, the epigenome. Also, while previous studies show that GSTP1 methylation decreases GSTP1 expression [19, 56], we were unable to measure GSPT1 expression due to logistical limitations that made collecting nasal cell RNA unfeasible. Future studies that include more direct measurement of oxidative stress could more definitively assess whether the observed associations are mediated through oxidative stress pathways. Given that our study examines these effects in an ethnically diverse population, additional studies are warranted to generalize our results to other populations. Finally, as our statistical modeling techniques evolve, it will be important to note that co-occurring pollutants may influence different periods of lung development and interact, thus highlighting the need for future multi-pollutant study.

In summary, we use data-driven statistical techniques to demonstrate that increased PM exposure during the final phase of gestation may increase risk for future impaired lung function, possibly mediated through epigenetic regulation of GSTP1.

Increased prenatal PM_2.5_ exposure in later pregnancy (≥ 35 weeks gestation) was associated with increased risk for impaired lung function in 7 year olds and increased GSTP1 methylation. Boys were especially susceptible. Elucidating timing of exposure may better inform mechanistic underpinnings and identify those at heightened risk.

## Conclusions

Data-drive statistical techniques demonstrate that prenatal PM_2.5_ exposure in late pregnancy was associated with impaired early childhood lung function and DNA hypermethylation of nasal epithelia GSTPI, especially in boys. Higher GSTP1 percent methylation was significantly associated with reduced FEV1. Future studies with larger sample size are needed to corroborate these findings and more formally examine mediation of the link between prenatal air pollution exposure and childhood lung function by GSTP1.

## Abbreviations

ACCESS: Asthma Coalition on Community, Environment and Social Stress
BDLIMs: Baysian distributed lag interaction models
CEE: Cumulative effect estimate
Cm: Centimeter
DIC: Deviance information criterion
FEF_25–75_: Forced expiratory flow between 25 and 75% of the FVC
FEV_1_: Forced expiratory volume in one second
FVC: Forced vital capacity
GSTP1: Glutathione S-transferase P1
Kg: Kilogram
NEC: Nasal epithelial cells
PCR: Polymerase chain reaction
PM_10_: Particulate matter with an aerodynamic diameter of less than 10 μm
PM_2.5_: Particulate matter with an aerodynamic diameter of less than 2.5 μm
ROS: Reactive oxygen species

## References

[1] Lange P, Celli B, Agusti A, Boje Jensen G, Divo M, Faner R, Guerra S, Marott JL, Martinez FD, Martinez-Camblor P (2015). Lung-function trajectories leading to chronic obstructive pulmonary disease. N Engl J Med.

[2] Vestbo J, Edwards LD, Scanlon PD, Yates JC, Agusti A, Bakke P, Calverley PM, Celli B, Coxson HO, Crim C (2011). Changes in forced expiratory volume in 1 second over time in COPD. N Engl J Med.

[3] Joad JP, Ji C, Kott KS, Bric JM, Pinkerton KE (1995). In utero and postnatal effects of sidestream cigarette smoke exposure on lung function, hyperresponsiveness, and neuroendocrine cells in rats. Toxicol Appl Pharmacol.

[4] Mauad T, Rivero DH, de Oliveira RC, Lichtenfels AJ, Guimaraes ET, de Andre PA, Kasahara DI, Bueno HM, Saldiva PH (2008). Chronic exposure to ambient levels of urban particles affects mouse lung development. Am J Respir Crit Care Med.

[5] Hsu HH, Chiu YH, Coull BA, Kloog I, Schwartz J, Lee A, Wright RO, Wright RJ (2015). Prenatal particulate air pollution and asthma onset in urban children. Identifying sensitive windows and sex differences. Am J Respir Crit Care Med.

[6] Jedrychowski WA, Perera FP, Maugeri U, Mroz E, Klimaszewska-Rembiasz M, Flak E, Edwards S, Spengler JD (2010). Effect of prenatal exposure to fine particulate matter on ventilatory lung function of preschool children of non-smoking mothers. Paediatr Perinat Epidemiol.

[7] Wilson A, Chiu YM, Hsu HL, Wright RO, Wright RJ, Coull BA (2017). Potential for Bias when estimating critical windows for air pollution in Children's health. Am J Epidemiol.

[8] Martino D, Prescott S (2011). Epigenetics and prenatal influences on asthma and allergic airways disease. Chest.

[9] Wright RJ (2010). Perinatal stress and early life programming of lung structure and function. Biol Psychol.

[10] Breton CV, Byun HM, Wenten M, Pan F, Yang A, Gilliland FD (2009). Prenatal tobacco smoke exposure affects global and gene-specific DNA methylation. Am J Respir Crit Care Med.

[11] Novakovic B, Ryan J, Pereira N, Boughton B, Craig JM, Saffery R (2014). Postnatal stability, tissue, and time specific effects of AHRR methylation change in response to maternal smoking in pregnancy. Epigenetics.

[12] Gruzieva O, Xu CJ, Breton CV, Annesi-Maesano I, Anto JM, Auffray C, Ballereau S, Bellander T, Bousquet J, Bustamante M (2017). Epigenome-wide meta-analysis of methylation in children related to prenatal NO2 air pollution exposure. Environ Health Perspect.

[13] Cai J, Zhao Y, Liu P, Xia B, Zhu Q, Wang X, Song Q, Kan H, Zhang Y (2017). Exposure to particulate air pollution during early pregnancy is associated with placental DNA methylation. Sci Total Environ.

[14] Li N, Kim S, Wang M, Froines J, Sioutas C, Nel A (2002). Use of a stratified oxidative stress model to study the biological effects of ambient concentrated and diesel exhaust particulate matter. Inhal Toxicol.

[15] Romieu I, Garcia-Esteban R, Sunyer J, Rios C, Alcaraz-Zubeldia M, Velasco SR, Holguin F (2008). The effect of supplementation with omega-3 polyunsaturated fatty acids on markers of oxidative stress in elderly exposed to PM(2.5). Environ Health Perspect.

[16] Alexis NE, Zhou H, Lay JC, Harris B, Hernandez ML, Lu TS, Bromberg PA, Diaz-Sanchez D, Devlin RB, Kleeberger SR, Peden DB (2009). The glutathione-S-transferase mu 1 null genotype modulates ozone-induced airway inflammation in humans. J Allergy Clin Immunol.

[17] Li YF, Gauderman WJ, Conti DV, Lin PC, Avol E, Gilliland FD (2008). Glutathione S-transferase P1, maternal smoking, and asthma in children: a haplotype-based analysis. Environ Health Perspect.

[18] Madrigano J, Baccarelli A, Mittleman MA, Wright RO, Sparrow D, Vokonas PS, Tarantini L, Schwartz J (2011). Prolonged exposure to particulate pollution, genes associated with glutathione pathways, and DNA methylation in a cohort of older men. Environ Health Perspect.

[19] Jhaveri MS, Morrow CS (1998). Methylation-mediated regulation of the glutathione S-transferase P1 gene in human breast cancer cells. Gene.

[20] de Planell-Saguer M, Lovinsky-Desir S, Miller RL (2014). Epigenetic regulation: the interface between prenatal and early-life exposure and asthma susceptibility. Environ Mol Mutagen.

[21] Spann K, Snape N, Baturcam E, Fantino E (2016). The impact of early-life exposure to air-borne environmental insults on the function of the airway epithelium in asthma. Ann Glob Health.

[22] Bergougnoux A, Claustres M, De Sario A (2015). Nasal epithelial cells: a tool to study DNA methylation in airway diseases. Epigenomics.

[23] McDougall CM, Blaylock MG, Douglas JG, Brooker RJ, Helms PJ, Walsh GM (2008). Nasal epithelial cells as surrogates for bronchial epithelial cells in airway inflammation studies. Am J Respir Cell Mol Biol.

[24] Thavagnanam S, Parker JC, McBrien ME, Skibinski G, Shields MD, Heaney LG (2014). Nasal epithelial cells can act as a physiological surrogate for paediatric asthma studies. PLoS One.

[25] Wright RJ, Suglia SF, Levy J, Fortun K, Shields A, Subramanian S, Wright R (2008). Transdisciplinary research strategies for understanding socially patterned disease: the asthma coalition on community, environment, and social stress (ACCESS) project as a case study. Cien Saude Colet.

[26] Kloog I, Chudnovsky AA, Just AC, Nordio F, Koutrakis P, Coull BA, Lyapustin A, Wang Y, Schwartz J (2014). A new hybrid spatio-temporal model for estimating daily multi-year PM2.5 concentrations across northeastern USA using high resolution aerosol optical depth data. Atmos Environ.

[27] Beydon N, Davis SD, Lombardi E, Allen JL, Arets HG, Aurora P, Bisgaard H, Davis GM, Ducharme FM, Eigen H (2007). An official American Thoracic Society/European Respiratory Society statement: pulmonary function testing in preschool children. Am J Respir Crit Care Med.

[28] Miller MR, Hankinson J, Brusasco V, Burgos F, Casaburi R, Coates A, Crapo R, Enright P, van der Grinten CP, Gustafsson P (2005). Standardisation of spirometry. Eur Respir J.

[29] Arets HG, Brackel HJ, van der Ent CK (2001). Forced expiratory manoeuvres in children: do they meet ATS and ERS criteria for spirometry?. Eur Respir J.

[30] Eigen H, Bieler H, Grant D, Christoph K, Terrill D, Heilman DK, Ambrosius WT, Tepper RS (2001). Spirometric pulmonary function in healthy preschool children. Am J Respir Crit Care Med.

[31] Morgan WJ, Stern DA, Sherrill DL, Guerra S, Holberg CJ, Guilbert TW, Taussig LM, Wright AL, Martinez FD (2005). Outcome of asthma and wheezing in the first 6 years of life: follow-up through adolescence. Am J Respir Crit Care Med.

[32] Lee AG, Chiu YM, Rosa MJ, Cohen S, Coull BA, Wright RO, Morgan WJ, Wright RJ (2017). Association of prenatal and early childhood stress with reduced lung function in 7-year-olds. Ann Allergy Asthma Immunol.

[33] Lai PS, Liang L, Cibas ES, Liu AH, Gold DR, Baccarelli A, Phipatanakul W (2015). Alternate methods of nasal epithelial cell sampling for airway genomic studies. J Allergy Clin Immunol.

[34] Barrow TM, Byun HM (2014). Single nucleotide polymorphisms on DNA methylation microarrays: precautions against confounding. Epigenomics.

[35] Baccarelli A, Wright RO, Bollati V, Tarantini L, Litonjua AA, Suh HH, Zanobetti A, Sparrow D, Vokonas PS, Schwartz J (2009). Rapid DNA methylation changes after exposure to traffic particles. Am J Respir Crit Care Med.

[36] Gasparrini A (2014). Modeling exposure-lag-response associations with distributed lag non-linear models. Stat Med.

[37] Morales E, Garcia-Esteban R, de la Cruz OA, Basterrechea M, Lertxundi A, de Dicastillo MD, Zabaleta C, Sunyer J (2015). Intrauterine and early postnatal exposure to outdoor air pollution and lung function at preschool age. Thorax.

[38] Bourbon J, Boucherat O, Chailley-Heu B, Delacourt C (2005). Control mechanisms of lung alveolar development and their disorders in bronchopulmonary dysplasia. Pediatr Res.

[39] Soler Artigas M, Loth DW, Wain LV, Gharib SA, Obeidat M, Tang W, Zhai G, Zhao JH, Smith AV, Huffman JE (2011). Genome-wide association and large-scale follow up identifies 16 new loci influencing lung function. Nat Genet.

[40] Cardenas A, Rifas-Shiman SL, Agha G, Hivert MF, Litonjua AA, DeMeo DL, Lin X, Amarasiriwardena CJ, Oken E, Gillman MW, Baccarelli AA (2017). Persistent DNA methylation changes associated with prenatal mercury exposure and cognitive performance during childhood. Sci Rep.

[41] Reik W (2007). Stability and flexibility of epigenetic gene regulation in mammalian development. Nature.

[42] Wright RJ, Brunst KJ (2013). Programming of respiratory health in childhood: influence of outdoor air pollution. Curr Opin Pediatr.

[43] Guarnieri M, Balmes JR (2014). Outdoor air pollution and asthma. Lancet.

[44] McCunney RJ (2005). Asthma, genes, and air pollution. J Occup Environ Med.

[45] Curjuric I, Imboden M, Schindler C, Downs SH, Hersberger M, Liu SL, Matyas G, Russi EW, Schwartz J, Thun GA (2010). HMOX1 and GST variants modify attenuation of FEF25-75% decline due to PM10 reduction. Eur Respir J.

[46] Fan XP, Ji XF, Li XY, Gao S, Fan YC, Wang K (2016). Methylation of the glutathione-S-transferase P1 gene promoter is associated with oxidative stress in patients with chronic hepatitis B. Tohoku J Exp Med.

[47] Dysart MM, Galvis BR, Russell AG, Barker TH (2014). Environmental particulate (PM2.5) augments stiffness-induced alveolar epithelial cell mechanoactivation of transforming growth factor beta. PLoS One.

[48] Tang W, Du L, Sun W, Yu Z, He F, Chen J, Li X, Li X, Yu L, Chen D (2017). Maternal exposure to fine particulate air pollution induces epithelial-to-mesenchymal transition resulting in postnatal pulmonary dysfunction mediated by transforming growth factor-beta/Smad3 signaling. Toxicol Lett.

[49] McCormack MC, Breysse PN, Matsui EC, Hansel NN, Williams D, Curtin-Brosnan J, Eggleston P, Diette GB (2009). In-home particle concentrations and childhood asthma morbidity. Environ Health Perspect.

[50] Wallace LA, Mitchell H, O'Connor GT, Neas L, Lippmann M, Kattan M, Koenig J, Stout JW, Vaughn BJ, Wallace D (2003). Particle concentrations in inner-city homes of children with asthma: the effect of smoking, cooking, and outdoor pollution. Environ Health Perspect.

[51] Wilson WE, Mage DT, Grant LD (2000). Estimating separately personal exposure to ambient and nonambient particulate matter for epidemiology and risk assessment: why and how. J Air Waste Manag Assoc.

[52] Rosa MJ, Jung KH, Perzanowski MS, Kelvin EA, Darling KW, Camann DE, Chillrud SN, Whyatt RM, Kinney PL, Perera FP, Miller RL (2011). Prenatal exposure to polycyclic aromatic hydrocarbons, environmental tobacco smoke and asthma. Respir Med.

[53] Miller MD, Marty MA (2010). Impact of environmental chemicals on lung development. Environ Health Perspect.

[54] Lee SY, Kim BS, Kwon SO, Oh SY, Shin HL, Jung YH, Lee E, Yang SI, Kim HY, Seo JH (2015). Modification of additive effect between vitamins and ETS on childhood asthma risk according to GSTP1 polymorphism: a cross -sectional study. BMC Pulm Med.

[55] Hsu WH, Hwang SA, Kinney PL, Lin S (2017). Seasonal and temperature modifications of the association between fine particulate air pollution and cardiovascular hospitalization in New York state. Sci Total Environ.

[56] Zhong S, Tang MW, Yeo W, Liu C, Lo YM, Johnson PJ (2002). Silencing of GSTP1 gene by CpG island DNA hypermethylation in HBV-associated hepatocellular carcinomas. Clin Cancer Res.

